# Splenic-vasculature involvement is associated with poor prognosis in resected distal pancreatic cancer

**DOI:** 10.1093/gastro/goaa084

**Published:** 2020-11-24

**Authors:** Feng Yin, Mohammed Saad, Jingmei Lin, Christopher R Jackson, Bing Ren, Cynthia Lawson, Dipti M Karamchandani, Belen Quereda Bernabeu, Wei Jiang, Teena Dhir, Richard Zheng, Christopher W Schultz, Dongwei Zhang, Courtney L Thomas, Xuchen Zhang, Jinping Lai, Michael Schild, Xuefeng Zhang, Hao Xie, Xiuli Liu

**Affiliations:** 1 Department of Pathology and Anatomical Sciences, University of Missouri, Columbia, MO, USA; 2 Department of Pathology, Indiana University, Indianapolis, IN, USA; 3 Department of Pathology, Dartmouth-Hitchcock Medical Center, Lebanon, NH, USA; 4 Department of Pathology, Pennsylvania State Health Milton S. Hershey Medical Center, Hershey, PA, USA; 5 Department of Pathology, Thomas Jefferson University Hospital, Philadelphia, PA, USA; 6 Department of Pathology and Laboratory Medicine, University of Rochester Medical Center, Rochester, NY, USA; 7 Department of Pathology, Yale University, New Haven, CT, USA; 8 Department of Pathology and Laboratory Medicine, Kaiser Permanente Sacramento Medical Center, Sacramento, CA, USA; 9 Department of Pathology, Duke University, Durham, NC, USA; 10 Department of Pathology, Cleveland Clinic, Cleveland, OH, USA; 11 Department of Gastrointestinal Oncology, Moffitt Cancer Center, Tampa, FL, USA; 12 Department of Pathology, Immunology and Lab Medicine, University of Florida, Gainesville, FL, USA

**Keywords:** pancreatic cancer, splenic artery, splenic vein, prognosis, intraductal papillary mucinous neoplasm

## Abstract

**Background:**

Distal pancreatic carcinoma is one of the most lethal cancers largely due to its high incidence of distant metastasis. This study aims to assess the prognostic value of splenic-vasculature involvement in resected distal pancreatic carcinoma.

**Methods:**

In this retrospective study, we collected the clinicopathologic information of 454 patients with pancreatic cancer and performed univariate and multivariate analyses to identify factors associated with progression-free survival (PFS) and overall survival (OS), with an emphasis on the prognostic value of splenic-artery and -vein involvement.

**Results:**

Univariate analysis revealed that larger tumor size, non-intraductal papillary mucinous neoplasm (non-IPMN)-associated adenocarcinoma, poor differentiation, stage pT3, nodal metastasis, lymphovascular invasion, perineural invasion, and pathologic and radiographic evidence of splenic-vein invasion were significantly associated with shorter PFS and OS (all *P* < 0.05). Multivariate analysis confirmed non-IPMN-associated adenocarcinoma, stage pT3, stage pN1–2, and post-operative adjuvant chemotherapy as independent risk factors for both PFS and OS, and larger tumor size and radiographic evidence of splenic-artery invasion as predictors of PFS only.

**Conclusion:**

Guidelines should be developed for a uniform approach with regard to the examination and reporting of the status of the splenic vasculature when dealing with distal-pancreatic-cancer specimens.

## Introduction

Pancreatic carcinoma is considered one of the most lethal cancers and is the fourth leading cause of cancer death in the USA [1]. The most common pancreatic cancer is pancreatic ductal adenocarcinoma. Patients with pancreatic adenocarcinoma located in the pancreatic body have a worse prognosis than those with cancer in the pancreatic head due to the inconspicuous early symptoms. Therefore, these tumors are frequently diagnosed at an advanced stage and curative surgery is usually only available for 10%–20% of the patients [2–4]. Unfortunately, even among patients with curative surgery, the 5-year survival rate is still low, ranging from 6% to 37% [5–7]. Local cancer recurrence and distant metastasis are the two most common causes of death. 

In 2017, the American Joint Committee Cancer (AJCC) 8th edition staging manual made significant changes to the pathological staging of pancreatic carcinomas [8]. Tumor size has become the major factor in defining pT1–3 pancreatic carcinoma, whereas carcinoma in the head of the pancreas with involvement of major large arteries including the superior mesenteric artery (SMA), common hepatic artery, or celiac axis has been classified as pT4 pancreatic cancer. Notably, the updates presented in AJCC 8th edition staging manual are largely based on clinical studies of carcinoma of the head of the pancreas. Whether the changes introduced in the latest AJCC 8th edition staging manual for pancreatic carcinomas applies to distal-pancreatic-cancer specimens (i.e. carcinoma of the pancreatic body and tail) remains unclear. 

Recent studies from Japanese groups have suggested that splenic-vasculature involvement is associated with a worse prognosis in distal pancreatic (body and tail) carcinoma [5, 9–11]. The effect of splenic-artery (SA) invasion in distal pancreatic cancer on disease-free survival was reported in a few reports [6]. In the Classification of Pancreatic Carcinoma 7th edition proposed by the Japanese Pancreas Society, pT3 was classified as ‘Tumor extends beyond the pancreas, but without involvement of celiac axis or superior mesenteric artery’ [12]. Specifically, SA involvement was considered tumor extension beyond the pancreas and was staged as pT3. Notably, there is a significant discordance of findings in terms of the clinical significance of splenic-vasculature involvement among these published studies. While some reports demonstrated that invasion of the SA, not the splenic vein (SV), was a significant prognostic factor [5, 6], the opposite conclusion was reached in some other studies [8, 10]. A relatively small study cohort among those studies and lack of histologic review of cases are the likely causes for this discrepancy. To address this significant clinical issue, we conducted a large-scale, multicentered study with histologic review of all cases to assess the prognostic value of splenic-vasculature involvement in resected distal pancreatic adenocarcinomas.

## Patients and methods

### Study design and study subjects

A retrospective study from seven US academic medical centers (University of Florida, Indiana University, Dartmouth-Hitchcock Medical Center, Penn State Health Milton S. Hershey Medical Center, Thomas Jefferson University Hospital, University of Rochester Medical Center, and Yale University) was performed. The research protocol was approved by the institutional review boards from individual institutions.

Patients who underwent radical resection of distal pancreatic ductal adenocarcinoma in the seven centers mentioned above between 2005 and 2018 were included. Patients with non-invasive intraductal papillary mucinous neoplasm (IPMN), non-invasive mucinous cystic neoplasm, and neuroendocrine neoplasms were excluded. Patients who had received preoperative neoadjuvant therapy were excluded from this study.

### Histopathology review

We carefully reviewed pathology reports and slides to obtain tumor information including tumor type, differentiation, site, size, margin status, lymph-node status, SA and SV involvement, and pathological staging. All tumors were restaged according to the AJCC 8th edition. SA involvement in this study was defined as invasion of the tumor into or through the arterial wall. SV involvement in this study was defined as invasion of the tumor through the venous wall.

Medical charts were reviewed from the time of resection until September 2018 to obtain the patients’ demographic data (age at resection and sex), radiographic data, recurrence, metastasis, and survival status. The interval between the date of surgery and the date of initial recurrence and/or metastasis of tumor or death was calculated as progression-free survival (PFS) and the interval between the date of surgery and the date of death was calculated as overall survival (OS).

### Statistical analysis

Continuous variables are presented as mean ± standard deviation and compared using the Wilcoxon rank-sum test. Categorical variables are expressed as count and percentage, and compared using the Fisher’s exact test. PFS and OS were estimated using Kaplan–Meier curves and compared using log-rank tests. Cox proportional-hazards models were used in univariate and multivariable survival analyses to identify factors associated with PFS and OS. The proportionality assumption was assessed graphically using log plots and quantitatively using the Z statistic. All tests were two-sided and performed in R (version 3.5.3). A *P*-value of <0.05 was considered statistically significant.

## Results

### Demographics and clinicopathologic characteristics

A total of 454 cases were included in the study, including 243 females (53.5%) and 211 males (46.5%), with a mean age of 67.1 years (range, 27–91 years). Radiographic findings associated with SA and SV involvement were reported in 73 (16.1%) and 93 (20.5%) cases, respectively, and pathologic diagnosis of SA and SV invasion were confirmed in 25 (5.5%) and 53 (11.7%) cases, respectively (Table 1). The overall SA and SV identification rate in pathology reports was 24.7% and 21.3% in distal-pancreatectomy specimens, respectively.

**Table 1.** Demographics and clinicopathologic features in 454 patients with distal pancreatic adenocarcinoma

Feature	No. of patients (%)
Age, years (mean ± SD)	67.1 ± 10.1
Sex	
Female	243 (53.5)
Male	211 (46.5)
Type of cancer	
Invasive IPMN	78 (17.2)
Non-IPMN-associated carcinoma	376 (82.8)
Tumor location	
Tail	229 (50.4)
Body	146 (32.2)
Tail/body	73 (16.1)
Tail/body/head	6 (1.3)
Histopathologic differentiation	
Well	41 (9.0)
Moderate	271 (59.7)
Poor	128 (28.2)
Unknown	14 (3.1)
T category, *n* (%)	
T1	76 (16.7)
T2	202 (44.5)
T3	166 (36.6)
Tx	10 (2.2%)
Lymph-node metastasis	
Positive	238 (52.4)
Negative	216 (47.6)
Post-operative adjuvant chemotherapy	
Yes	84 (18.5)
No	303 (66.7)
Unknown	67 (14.8)
Pathologic SA invasion	
Present	25 (5.5)
Absent	249 (54.8)
Not reported	180 (39.6)
Pathologic SV invasion	
Present	53 (11.7)
Absent	220 (48.5)
Not reported	181 (39.9)
Splenic parenchymal invasion	
Present	25 (5.5)
Absent	425 (93.6)
Not reported	4 (0.9)
Radiographic SA invasion	
Present	73 (16.1)
Absent	242 (53.3)
Not examined	139 (30.6)
Radiographic SV invasion	
Present	93 (20.5)
Absent	228 (50.2)
Not examined	133 (29.3)

IPMN, intraductal papillary mucosal neoplasm; SA, splenic artery; SV, splenic vein; Tx, pathologic tumor stage not specified.

### Prognostic factors

In univariate analysis, factors such as sex, age, tumor location, proximal pancreatic margin involvement, anterior pancreatic surface and posterior margin involvement, and adjuvant radiation therapy were associated neither with PFS nor with OS significantly, whereas tumor size, diagnosis group (IPMN vs non-IPMN), poor differentiation, stage T3, positive lymph-node metastasis, lymphovascular invasion, perineural invasion, pathologic SV invasion, and radiographic evidence of SV invasion were significantly associated with shorter PFS and OS (all *P* < 0.05; Table 2). Post-operative adjuvant chemotherapy was associated with shorter PFS (*P* = 0.02) and longer OS (*P* < 0.001). Interestingly, pathologic SA invasion (*P* = 0.05), splenic parenchymal invasion (*P* = 0.009), and radiographic evidence of SA invasion (*P* = 0.03) were only significantly associated with PFS, but not with OS.

**Table 2.** Univariate analysis for survival in 454 patients with distal pancreatic adenocarcinoma

Variable	Progression-free survival		Overall survival	
	HR (95% CI)	*P*-value	HR (95% CI)	*P*-value
Sex (male vs female)	1.01 (0.77–1.32)	0.94	1.10 (0.88–1.37)	0.39
Age (every 1-year increase)	0.99 (0.98–1.00)	0.07	1.01 (1.00–1.02)	0.13
Tumor size (every 1-cm increase)	1.11 (1.04–1.18)	0.001	1.10 (1.04–1.15)	<0.001
Diagnosis group (non-IPMN vs IPMN)	2.62 (1.71–4.02)	<0.001	1.94 (1.41–2.66)	<0.001
Differentiation				
Well	Reference	–	Reference	
Moderate	1.46 (0.89–2.39)	0.14	1.26 (0.83–1.90)	0.28
Poor	1.75 (1.03–2.96)	0.04	1.88 (1.22–2.90)	0.004
T category				
T1	Reference	–	Reference	–
T2	1.97 (1.27–3.05)	0.002	1.26 (0.90–1.75)	0.18
T3	2.27 (1.45–3.56)	<0.001	1.75 (1.25–2.46)	0.001
N category				
N0	Reference	–	Reference	–
N1	1.93 (1.45–2.58)	<0.001	1.78 (1.40–2.27)	<0.001
N2	1.98 (1.30–3.02)	0.001	2.04 (1.46–2.84)	<0.001
Lymphovascular invasion (small vessel)	1.65 (1.25–2.17)	<0.001	1.49 (1.19–1.87)	<0.001
Perineural invasion	1.55 (1.08–2.23)	0.02	1.60 (1.18–2.18)	0.003
Pathologic SA invasion	1.81 (0.99–3.29)	0.05	1.44 (0.89–2.31)	0.13
Pathologic SV invasion	1.93 (1.23–3.04)	0.004	2.21 (1.57–3.11)	<0.001
Splenic parenchymal invasion	2.02 (1.19–3.41)	0.009	1.40 (0.89–2.20)	0.15
Adjuvant radiation therapy	0.98 (0.74–1.28)	0.86	0.86 (0.67–1.10)	0.23
Adjuvant chemotherapy	1.68 (1.09–2.59)	0.02	0.57 (0.43–0.75)	<0.001
Radiographic SA invasion	1.47 (1.04–2.07)	0.03	1.25 (0.91–1.71)	0.17
Radiographic SV invasion	1.45 (1.05–2.01)	0.02	1.55 (1.16–2.08)	0.003

IPMN, intraductal papillary mucinous neoplasms; SA, splenic artery; SV, splenic vein; HR, hazard ratio; CI, confidence interval.

In multivariable analysis, diagnosis group (hazard ratio [HR] = 2.47, *P* = 0.01 for PFS and HR = 2.20, *P* = 0.012 for OS), stage N1 (HR = 2.12, *P* = 0.006 for PFS and HR = 2.75, *P* < 0.001 for OS), and adjuvant chemotherapy (HR = 3.64, *P* = 0.007 for PFS and HR = 0.49, *P* = 0.005 for OS) were independent prognostic factors for both PFS and OS, whereas tumor size (HR = 1.28, *P* = 0.020) and radiographic evidence of SA invasion (HR = 4.07, *P* = 0.005) were independent prognostic factors for PFS only (Table 3).

**Table 3.** Multivariate analysis for survival in 454 patients with distal pancreatic adenocarcinoma

Variable	Progression-free survival		Overall survival	
	HR (95% CI)	*P*-value	HR (95% CI)	*P*-value
Tumor size (every 1-cm increase)	1.28 (1.04–1.58)	0.02	1.11 (0.92–1.33)	0.26
Diagnosis group (non-IPMN vs IPMN)	2.47 (1.23–4.96)	0.01	2.20 (1.19–4.07)	0.01
Differentiation				
Well	Reference	–	Reference	–
Moderate	1.34 (0.59–3.07)	0.49	1.25 (0.59–2.65)	0.56
Poor	1.15 (0.48–2.80)	0.75	1.60 (0.73–3.51)	0.24
T category				
T1	Reference	–	Reference	–
T2	0.99 (0.47–2.06)	0.97	0.89 (0.45–1.76)	0.73
T3	0.63 (0.19–2.07)	0.45	0.62 (0.21–1.81)	0.38
N category				
N0	Reference	–	Reference	–
N1	2.12 (1.24–3.63)	0.006	2.75 (1.59–4.73)	<0.001
N2	2.04 (0.82–5.12)	0.13	2.68 (1.21–5.95)	0.015
Lymphovascular invasion (small vessel)	0.77 (0.43–1.38)	0.39	0.83 (0.49–1.42)	0.50
Perineural invasion	1.13 (0.61–2.09)	0.69	1.13 (0.64–1.98)	0.67
Pathologic SA invasion	1.13 (0.43–2.95)	0.80	–	–
Pathologic SV invasion	1.98 (0.83–4.71)	0.12	1.66 (0.88–3.15)	0.12
Splenic parenchymal invasion	1.12 (0.32–3.92)	0.86	–	–
Adjuvant chemotherapy	3.64 (1.41–9.38)	0.007	0.49 (0.30–0.80)	0.005
Radiographic SA invasion	4.07 (1.51–10.97)	0.005	–	–
Radiographic SV invasion	0.37 (0.14–1.02)	0.05	1.12 (0.70–1.80)	0.64

IPMN, intraductal papillary mucosal neoplasms; SA, splenic artery; SV, splenic vein; HR, hazard ratio; CI, confidence interval.

### Association of splenic-vasculature involvement with survival

**Figure 1.**
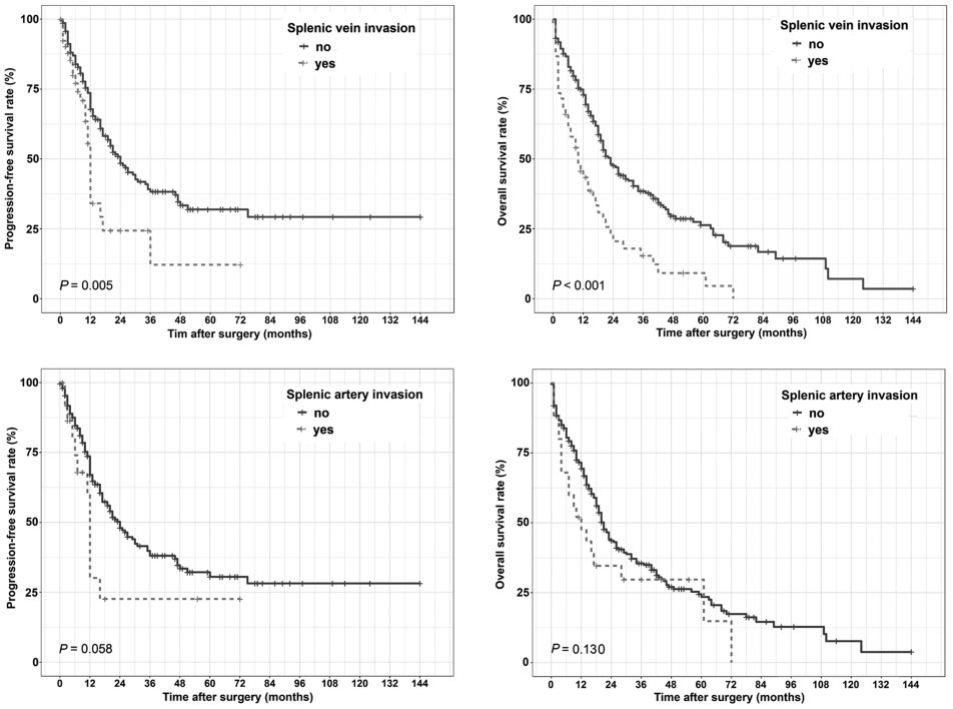
Comparisons of progression-free survival and overall survival in patients with or without invasion of the splenic artery or the splenic vein.

As shown in Figure 1, the median OS of 53 patients with SV invasion and 220 patients without SV invasion were 10 and 23 months, respectively, and the difference was statistically significant (*P* < 0.001). The median time to relapse or metastasis among patients with SV invasion was also significantly shorter than that for those without SV invasion (12 vs 24 months, *P* = 0.005). On the other hand, there were no statistically significant differences between patients with SA invasion and those without SA invasion with regard to OS and PFS (OS, *P* = 0.130; PFS, *P* = 0.058).

### Inter-institutional variabilities in macroscopic assessment of the splenic vasculature

The inter-institutional variabilities in macroscopic examination of splenic vasculature and surface inking in resected distal-pancreatectomy specimens were also evaluated in the study. Among the seven academic medical centers, the mean reporting rates for SA and SV in pathology reports were 24.7% (range, 6.9%–36.4%) and 21.4% (range, 6.9%–28.8%), respectively. The mean reported inking rates for anterior pancreatic surface and posterior pancreatic margin were 82.4% (range, 46.2%–100%) and 86.8% (range, 47.7%–100%), respectively (Table 4).

**Table 4.** Inter-institutional variabilities in the documentation of macroscopic examinations

Institution	No. of cases	SA reporting (%)	SV reporting (%)	AS inking (%)	PS inking (%)
Institution A	154	43 (27.9)	38 (24.7)	151 (98.1)	152 (98.7)
Institution B	66	24 (36.4)	19 (28.8)	30 (45.5)	31 (47.0)
Institution C	36	4 (11.1)	4 (11.1)	36 (100.0)	36 (100.0)
Institution D	76	16 (21.1)	19 (25.0)	66 (86.8)	66 (86.8)
Institution E	58	4 (6.9)	4 (6.9)	30 (51.7)	48 (82.8)
Institution F	31	9 (29.0)	6 (19.4)	31 (100.0)	31 (100.0)
Institution G	33	12 (36.4)	7 (21.2)	30 (90.9)	30 (90.9)
P-value		0.001	0.038	<0.001	<0.001

SA, splenic artery; SV, splenic vein; AS, anterior pancreatic surface; PS, posterior surface/margin.

## Discussion

The study demonstrated multiple independent prognostic factors for patients with resectable distal pancreatic adenocarcinoma, including tumor size, diagnosis group, and pathologic T/N stage. In this study, post-operative adjuvant chemotherapy was associated with longer OS, consistently with the literature [13, 14]. However, a shorter PFS was noted in patients receiving post-operative adjuvant chemotherapy, indicating an underlying higher risk of recurrence in patients receiving adjuvant chemotherapy. Preoperative neoadjuvant therapy with subsequent surgery is one of the standard treatment strategies for pancreatic cancer. A recent study showed that preoperative gemcitabine-based chemoradiation therapy contributed to a favorable 5-year survival outcome of the resected cases and SA invasion was associated with poor survival due to the greater incidence of distant recurrence [15]. However, as a potential confounding factor through its ‘tumor-downstage’ effect, the patients receiving preoperative neoadjuvant therapy were excluded from the current study. In this study, we specifically examined the prognostic impact of the splenic-vascular invasion in a large US cohort of distal pancreatic adenocarcinoma for the first time.

The pancreas lies on several major vascular structures. The aorta and inferior vena cava are located posterior to the pancreatic head and the SMA is located anterior to the uncinate process. The current AJCC 8th edition staging manual defines pT4 carcinoma solely based upon the involvement of these major arteries. Due to the anatomic location, pancreatic body and tail tumors rarely involve these major arteries. Instead, the SA (a branch from the celiac axis) and the SV (drain to the hepatic portal vein) become the major vasculatures for local extension in these cases. Several recent studies have been conducted to explore the prognostic impact of the SA and the SV in patients with pancreatic ductal adenocarcinoma, but the results are controversial. Kanda *et al*. [5] and Partelli *et al.* [6] proposed that invasion of the SA, but not the SV, is a significant poor prognostic factor. SA invasion is not an independent prognostic factor in a study from Fukami’s group [9]. On the contrary, Shimada *et al.* [10] and Mizumoto *et al.* [11] showed that invasion of the SV, but not the SA, is an independent predictor of poor prognosis in pancreatic adenocarcinoma of the body and tail. The obvious discrepancy from those studies is largely unclear, but most of those studies were conducted with relatively small patient cohorts ranging from 51 to 87 patients who underwent distal pancreatectomy. Another potential reason for this discrepancy is that the SA and SV involvement in some studies was defined as invasion into but not through the vessel wall. A recent meta-analysis study based on these reports has concluded that both SA and SV invasions are associated with poor survival in patients with resectable pancreatic cancer [16]. To clarify this important issue, we conducted this multi-institutional large-scale study and our results verified radiographic evidence of SA invasion as an independent prognostic factor for PFS, but not for OS. A recent study also demonstrated a poor prognosis in distal-pancreatic-cancer patients presenting with radiographic evidence of SA invasion, although, in that study, radiographic SA invasion was identified as an independent risk factor for OS [17]. The underlying cause of this discrepancy is unclear; however, even though the median OS for the entire cohort in our study was similar to this published study (18 vs 21 months), the identification rate for radiographic SA invasion in our study was much lower than that in this published study (16% vs 41%). Interestingly, pathologic evidence of SA invasion is not extracted as an independent prognostic factor. This may be due to the fact that more cases were examined for radiographic evidence of SA invasion other than for histological evidence of SA invasion in our study. It should be noted that pathologic evidence of SV invasion is associated with poor prognosis by univariate analysis.

Based on our study, the radiographic and pathological evidence of splenic-vasculature involvement should be examined carefully pre and post-operatively, as they are prognostic factors for survival. However, due to the overall low frequency of the distal-pancreatectomy specimens, a uniform approach has not been consistently adopted for accurately documenting splenic-vasculature invasion in pathology reports. Indeed, our study demonstrated that the splenic-vascular identification rate was ∼20% in distal-pancreatectomy specimens and varied significantly among academic centers. The results from our study suggest that guidelines should be developed for a uniform approach to facilitate the identification, examination, and sampling of the splenic vasculature in order to adequately document the tumor invasion of these structures—an independent predictor of poor prognosis in distal pancreatic cancers. Similarly, a standard radiographic protocol should be developed and used preoperatively to assess splenic-vasculature involvement in patients with distal pancreatic cancers.

Our study has several strengths. It is the largest study to have examined the pathological parameters in distal pancreatic cancers. In addition, all cases were reviewed by pathologists with an interest and expertise in pancreatic cancer. Further, the prognostic role of splenic-vasculature involvement was performed in this study along with other important factors such as adjuvant therapy, which was not included in previous studies for disease-free survival. In addition, all tumors were restaged according to the AJCC 8th edition. Even with relatively strict selecting criteria, wide heterogeneity of diagnostic groups is still inevitable in our study objects. For example, IPMN and non-IPMN-associated adenocarcinoma in our study had different underlying tumorigenesis and clinical behavior [18–20]. The reporting rate of splenic-vasculature involvement either in radiographic or surgical pathology diagnosis is disappointingly low. In addition, all cases were from large cancer centers, which may have introduced bias. Furthermore, the cases were from a 13-year period during which previous AJCC staging editions (the 5th, 6th, and 7th) had been used for staging pancreatic cancers.

In summary, our study demonstrated that splenic-vasculature invasion was associated with poor survival in distal pancreatic cancer in addition to tumor size, diagnostic group (invasive IPMN vs non-IPMN-associated adenocarcinoma), T staging, N metastases, and adjuvant chemotherapy—the known prognostic factors in pancreatic cancers. Our results suggest that invasion of the splenic vessel should be reported in the pathology report, as in the case of the radiographic report. We believe that the data generated by this uniform approach will become available in the near future to provide more evidence for whether this should be incorporated into a future AJCC staging system for distal pancreatic cancers.
